# Antiviral effects of deoxynojirimycin (DNJ)-based iminosugars in dengue virus-infected primary dendritic cells

**DOI:** 10.1016/j.antiviral.2022.105269

**Published:** 2022-03

**Authors:** Nilanka Perera, Juliane Brun, Dominic S. Alonzi, Beatrice E. Tyrrell, Joanna L. Miller, Nicole Zitzmann

**Affiliations:** a Department of Biochemistry, University of Oxford, Oxford, OX1 3QU, UK; b Department of Medicine, Faculty of Medical Sciences, University of Sri Jayewardenepura, Nugegoda, Sri Lanka

**Keywords:** α-glucosidases, Antiviral effects, Dendritic cells, Dengue, Iminosugars

## Abstract

Dendritic cells (DCs) are important targets for dengue virus (DENV) infection and play a significant role in the early immune response. Antiviral effects of iminosugars against DENV in primary cells have been demonstrated previously in monocyte-derived macrophages (MDMΦs). Given the important role played by DCs in innate immune defense against DENV, the antiviral effects of three deoxynojirimycin (DNJ) derivatives (*N*N-DNJ, E*O*O-DNJ and 2THO-DNJ) and a deoxygalactonojirimycin (DGJ) negative control were evaluated in DENV-infected primary human monocyte-derived immature DCs (imDCs). DNJ- but not DGJ-derivatives elicited antiviral activity in DENV-infected imDCs, similar to that observed in MDMΦs. The DNJ-derivatives inhibited DENV secretion in a dose-dependent manner. Endoplasmic reticulum (ER) α-glucosidase I inhibition by DNJ-derived iminosugars, at concentrations of 3.16 μM, correlated with a reduction in the specific infectivity of virions that were still secreted, as well as a reduction in DENV-induced tumour necrosis factor alpha secretion. This suggests iminosugar-mediated ER α-glucosidase I inhibition may give rise to further benefits during DENV infection, beyond the reduction in viral secretion associated with ER α-glucosidase II inhibition.

## Highlights

• DNJ-derived iminosugars *N*N-DNJ, 2THO-DNJ and E*O*O-DNJ reduce secretion of DENV from infected human monocyte-derived DCs.
• Specific infectivity of DC-produced DENV is reduced by DNJ-derived iminosugars at concentrations inhibiting ER α-glucosidase I.
• Secretion of TNFα by DENV-infected DCs is reduced by iminosugars at concentrations inhibiting ER α-glucosidase I.

## Abbreviations

ADE: Antibody dependent enhancement
αGluI: α-glucosidase I
αGluII: α-glucosidase II
2THO-DNJ: 8-tetrahydrofuranyl-octyl-deoxynojirimycin
BSA: Bovine serum albumin
CC_50_: concentration at which cell viability is 50%
DC: Dendritic cell
DC-SIGN: Dendritic cell-specific ICAM-3 grabbing non-integrin
DENV: Dengue virus
DNJ: Deoxynojirimycin
E*O*O-DNJ: *N*- (8′-ethoxyoctyl)-deoxynojirimycin
ER: Endoplasmic reticulum
FOS: Free oligosaccharides
HIV: Human immunodeficiency virus
HPLC: High-performance liquid chromatography
IC_50,_ IC_90_: 50% and 90% inhibitory concentrations
imDC: Immature dendritic cell
MDMΦs: Monocyte-derived macrophages
MOI: Multiplicity of infection
M*O*N DNJ: *N*‐(9‐methoxynonyl)‐1‐deoxynojirimycin
MR: Mannose receptor
*N*N-DGJ: *N*-(n-nonyl)-deoxygalactonojirimycin
*N*N-DNJ: *N*-(n-nonyl)-deoxynojirimycin
PBS: Phosphate buffered saline
TNFα: Tumour necrosis factor alpha

## 1 Introduction

Dengue virus (DENV) infections cause significant morbidity and mortality with increasing global incidence (Bhatt et al., 2013). While there has been recent progress with vaccine development (Wilder-smith, 2020), the quest for an effective antiviral continues. Certain iminosugars have long been under investigation as antiviral agents against several enveloped viruses (Zitzmann et al., 1999; Wu et al., 2002; Dwek et al., 2002), and previous studies have demonstrated that deoxynojirimycin (DNJ)-derived iminosugars reduce DENV secretion in a variety of infected cultured cells (Wu et al., 2002; Warfield et al., 2015). We have shown that DNJ-derived iminosugars have anti-DENV effects in primary monocyte derived macrophages (MDMΦs) and inhibit pathogen-induced inflammatory responses (Sayce et al., 2021). Promising *in vitro* and *in vivo* data for iminosugar antiviral activity against DENV have paved the way for iminosugars to enter clinical trials.

DNJ-derived iminosugars are glucose mimetics which possess a nitrogen atom in place of the oxygen in the carbohydrate ring; these inhibit host α-glucosidases pivotal to endoplasmic reticulum (ER) glycoprotein folding quality control (Alonzi et al., 2017). The outer α-1,2-linked glucose is trimmed by ER α-glucosidase I (αGluI) and the inner α-1,3-linked glucoses are removed by ER α-glucosidase II (αGluII) during N-linked glycan processing (Chang et al., 2013). Impaired glucose trimming due to iminosugar treatment results in misfolded proteins which are either degraded by ER-associated degradation or secreted with altered properties. Iminosugar treatment of virally infected cells thus disrupts proper virion morphogenesis (Alonzi et al., 2017). Though iminosugars can inhibit both α-glucosidases and glycolipid processing pathways, previous work has shown that inhibition of α-glucosidases underlies the antiviral effect of iminosugars in DENV infection in MDMΦs (Sayce et al., 2016; Miller et al., 2018). While iminosugars inhibit αGluII at lower concentrations and αGluI at higher concentrations of compound, inhibition of αGluII alone leads to measurable anti-flaviviral activity (Kiappes et al., 2018). However, the potentially enhanced effects of combined αGluI and αGluII inhibition seen with higher iminosugar concentrations have not been fully explored in DENV infection.

Dendritic cells (DCs) play a crucial role in combating infection by diverse pathogens (Cheong et al., 2011; Nakano et al., 2009; Schmid and Harris, 2014) and DCs are the first cells to encounter the virus following a mosquito bite in DENV infection (Marovich et al., 2001). Tumour necrosis factor alpha (TNFα), secreted by immune cells including DCs (Ho et al., 2001), is an important mediator in the development of severe disease and higher levels have been identified in patients with dengue haemorrhagic fever and dengue shock syndrome compared with dengue fever (Hober et al., 1996; Green et al., 1999; Gagnon et al., 2002). TNFα secreted from DENV-infected immune cells leads to fluid leakage by activating vascular endothelial cells (Anderson et al., 1997), it activates the coagulation cascade (Hober et al., 1993) and induces production of vasoactive substances (Anderson et al., 1997).

The effect of DNJ-derived iminosugars on DENV-infected primary DCs has not yet been investigated. In addition, the effect of DNJ-derived iminosugars on TNFα secretion by DENV-infected DCs is yet to be explored. This study examined the antiviral effects of iminosugar treatment on DENV-infected immature dendritic cells (imDCs), as well as the impact on TNFα secretion.

## 2 Materials and methods

### 2.1 Viruses and cells

DENV2 strain 16681 (a gift from G. Screaton, Oxford, UK) was propagated in the C6/36 insect cell line (a gift from the Armed Forces Research Institute of Medical Sciences, Thailand). Human monocytes were isolated from buffy coats (NHS Blood and Transplant, surplus to clinical requirements) as described previously (Miller et al., 2008) and cultured in RPMI-1640 media (with 10% heat-inactivated foetal bovine serum) for 4 days, supplemented with 50 ng/ml IL-4 and 100 ng/ml granulocyte colony stimulating factor (GM-CSF, Peprotech) to differentiate the cells into imDCs. The use of human blood was approved by the NHS National Research Ethics Service (09/H0606/3). Flow cytometry was performed to confirm the phenotype of imDCs. Cells were scraped from the flasks, washed in cold PBS, re-suspended in FACS block (0.5%w/v BSA, 5 mM EDTA in PBS, 5%v/v heat inactivated human serum) and placed on ice for 30 min and stained with primary antibody for 45 min on ice. 10 μg/ml anti-DC-SIGN (R&D, #MAB161) and 10 μg/ml anti-CD86 (BioLegend, #305401) antibodies were compared to 10 μg/ml IgG2b (R&D, #MAB004) isotype antibody. 50 μg/ml anti-CD14 (R&D, #MAB3832), 20 μg/ml anti-CD16 (BioLegend, #305301), 10 μg/well anti-CD83 (BioLegend, #305307) and 20 μg/ml anti-MR antibodies (clone 3.29.B1; a gift from Luisa Martinez-Pomares, University of Nottingham) were compared to 50 μg/ml IgG1 (clone D1.3) isotype control. Cells were re-suspended in 2 μg/ml secondary antibody (PE-Goat anti-mouse IgG (F(ab’)2 (eBioscience)) after two washes with FACS buffer and kept on ice for 45 min. Cells were subjected to two further washes before re-suspending in FACS buffer. Data were collected using a FACS Calibur machine (BD Biosciences) with approximately 10,000 gated cells per population and analysed using BD CellQuest™ software. Histograms were used to determine the expression of the surface receptors by comparing staining with target antibodies with the relevant isotype control.

### 2.2 Iminosugar derivatives

**Fig. 1:**
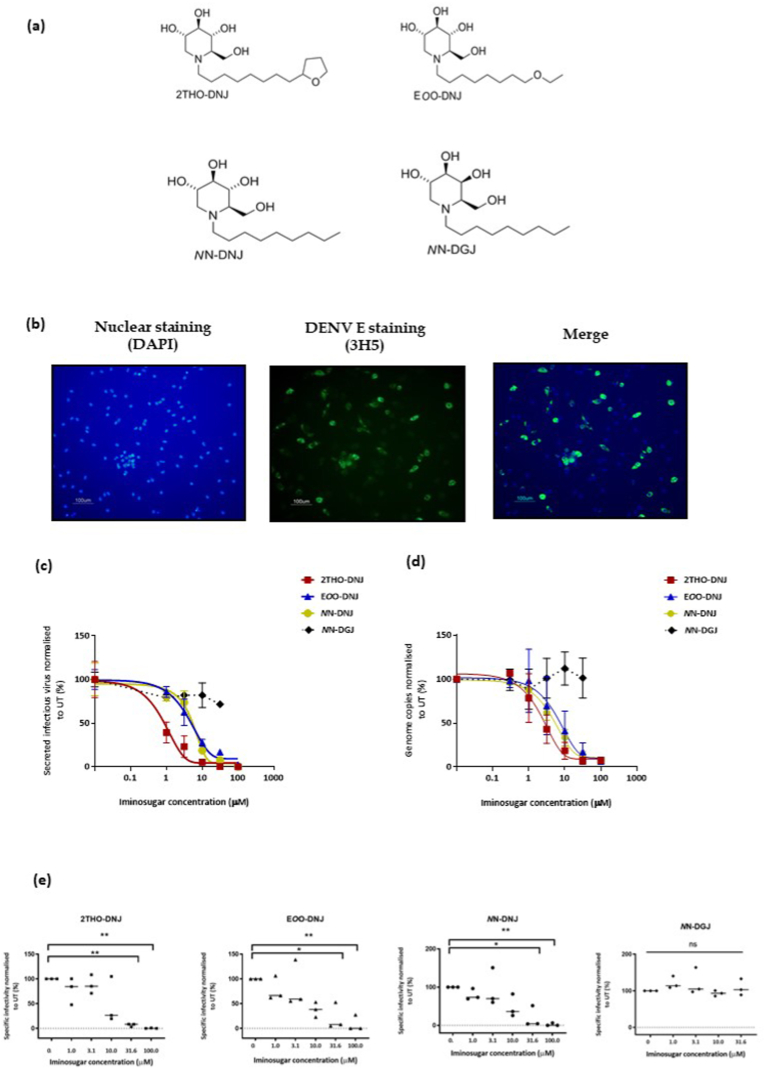
**Infection of imDCs by DENV and inhibition of infection by iminosugars.** (a) Chemical structures of the iminosugars used for the experiments. (b) Primary human imDCs matured from peripheral blood-derived monocytes were infected with DENV at a multiplicity of infection (MOI) of 1 for 48 h before the cell supernatant was removed and stored at −80 °C and the cells fixed, stained and visualised by fluorescent microscopy. The imDCs expressed the DENV envelope protein detected by the 3H5 antibody (green) and nuclei stained by DAPI (blue). DENV-infected imDCs were treated with serial titration of iminosugars for 48 h before the cell supernatant was assayed for DENV. (c) Infectious virus was quantified by a plaque assay and (d) total DENV genome copies by qRT-PCR. All data are normalized to the untreated (UT) DENV-infected control. Values represent mean ± SEM. (e) Scatter dot plots show specific infectivity of the secreted virus, calculated in UT and iminosugar-treated DENV-infected imDCs. The line represents the median. Data are derived from 3 independent experiments. *p < 0.05; **p < 0.01; ns, not significant.

Three DNJ-derived iminosugars with different alkyl chain compositions were studied: 2THO-DNJ (8-tetrahydrofuranyl-octyl-deoxynojirimycin, kindly given by Emergent BioSolutions Ltd), E*O*O-DNJ (*N*- (8′-ethoxyoctyl)-deoxynojirimycin, Oxford Glycobiology Institute) and *N*N-DNJ (*N*-(n-nonyl)-deoxynojirimycin, a gift from Oxford GlycoSciences Ltd). The galactose analogue-containing deoxygalactonojirimycin (DGJ)-derivative, *N*N-DGJ (purchased from Toronto Research Chemicals), was used as a control in these experiments as an iminosugar that does not inhibit α-glucosidases (Fig. 1a). *N*N-DNJ, *N*N-DGJ and 2THO-DNJ were solubilized in water, while E*O*O-DNJ was solubilized in DMSO. All iminosugar compounds tested were confirmed to have less than 0.05 endotoxin units/mL.

### 2.3 Cytotoxicity assay

Cytotoxicity of iminosugars was assessed by assaying for metabolic activity using a CellTiter 96® AQueous One Solution Cell Proliferation Assay (Promega) as per the manufacturer's instructions. Samples were incubated for 1–4 h (37 °C, 5% CO_2_) and the absorbance was measured at 490 nm (A_490_) on a SpectraMax M5 microplate reader (Molecular Devices). Absorbance was normalized to untreated controls after subtracting the blank reading. CC_50_ was defined as concentration of iminosugar at which cell viability was 50%.

### 2.4 Antiviral assays on imDCs

ImDCs were infected with DENV2 strain 16681 at a multiplicity of infection (MOI) of 1 for 90 min at room temperature, then virus containing supernatant was replaced with RPMI-1640 medium (Cambrex) supplemented with 10% foetal bovine serum and serial dilutions of iminosugar. Cells were incubated for 48 h at 37 °C with 5% CO_2_. Then the cell culture supernatant was removed, centrifuged for 5 min at 400×*g* to pellet any cells, and stored at −80 °C. Cells were fixed with 4% paraformaldehyde and permeabilised.

Infectious viral titers were obtained by viral plaque assay (Miller et al., 2008) on LLC-MK_2_ monkey kidney cells (limit of plaque detection of 33 plaque forming units [PFU]/ml). The percentage of infected cells was measured by immunofluorescence (Miller et al., 2008) using monoclonal antibody 3H5, specific for DENV2 envelope protein. The 50% and 90% inhibitory concentrations (IC_50_ and IC_90,_ respectively) were calculated based on experiments with imDCs derived from at least three donors. The selectivity index (SI) was calculated as follows: SI = CC_50_/IC_50_.

Total secreted viral genomes were quantified by extracting viral RNA from cell culture supernatant using the Directzol RNA mini prep kit (Zymo Research). A one-step quantitative reverse transcription polymerase chain reaction (qRT-PCR) for DENV NS5 was performed using the Thermo Scientific Verso one–step RT qPCR kit. A 2 μL volume of total template RNA in a final 20 μL reaction was run at 48 °C for 30 min (reverse transcription), 95 °C for 15 min (initial denaturation) followed by 40 cycles of 95 °C for 15 s, 55 °C for 60 s and 60 °C for 25 s before reading the plate. All samples were run in technical duplicates and compared to a standard curve of high titre viral RNA isolated from C6/36-grown DENV-2 for quantification. Forward (3′-ACAAGTCGAACAACCTGGTCC) and reverse (3′-GCCGCACCATTGGTCTTCTC) primers for dengue NS5 were used. The qRT-PCR was performed using an Applied Biosystems 7500 Fast Real-Time PCR System. Specific infectivity was calculated as follows: Specific infectivity = infectious virus in pfu/total viral genome copies. The value was normalized to the untreated (no iminosugar) control.

### 2.5 Functional TNFα assay

Functional TNFα levels were measured using HEK-Blue™ TNFα reporter cells (Invivogen, catalogue #hkb-tnfdmyd) cultured per manufacturer's instructions. Stimulation of these cells by TNFα results in activation of the NF-κB inducible promoter and production of secreted alkaline phosphatase (SEAP). To detect biologically functional TNFα, reporter cells were stimulated with cell culture supernatants from DENV-infected and/or iminosugar-treated imDCs for 24 h. SEAP secretion was quantified using QUANTI-Blue™ (Invivogen) as per manufacturer's instructions, by measuring absorbance at 645 nm (A_645_) using a NOVOstar microplate reader (BMG Labtech). Comparison with a standard curve of human recombinant TNFα (Peprotech, catalogue # 300-01A) was used to calculate the functional cytokine levels in the cell culture supernatant.

### 2.6 Detection of free oligosaccharides

Free oligosaccharides (FOS) accumulate due to inhibition of the ER α-glucosidases and quantification of the exact type of FOS provides an indication of αGluI and αGluII inhibition *in vitro*. Uninfected imDCs or imDCs infected with DENV2 at MOI 1 were treated with 0.3, 3.16 or 31.6 μM concentrations of iminosugars or left untreated. After 48 h post-infection, cells were lysed and analysed by normal phase (NP)-high-performance liquid chromatography (HPLC) for detection of FOS (Miller et al., 2008). For enzymatic determination of individual glucose-terminating and galactose-terminating FOS species, samples were subjected to digestion with β-galactosidase at 50 U/ml at 37 °C overnight to remove the terminal galactose residues. Peak area was used to assess molar quantity in comparison to standards of known identity and quantity. All FOS values were normalized based on the protein concentration.

### 2.7 Statistical analyses

Data were analysed using GraphPad Prism version 9.2.0 (GraphPad Software, Inc). For plaque assay data, the IC_50_ was calculated by fitting a logarithmic four-point sigmoidal curve to each data set. One-way ANOVA with Dunnett's multiple comparisons test was used to compare functional TNFα data among iminosugar treatments. Mean values for each set of biological replicates are given in figures with error bars representing standard error of the mean (SEM).

## 3 Results

### 3.1 Primary monocyte-derived imDC model

**Fig. A1:**
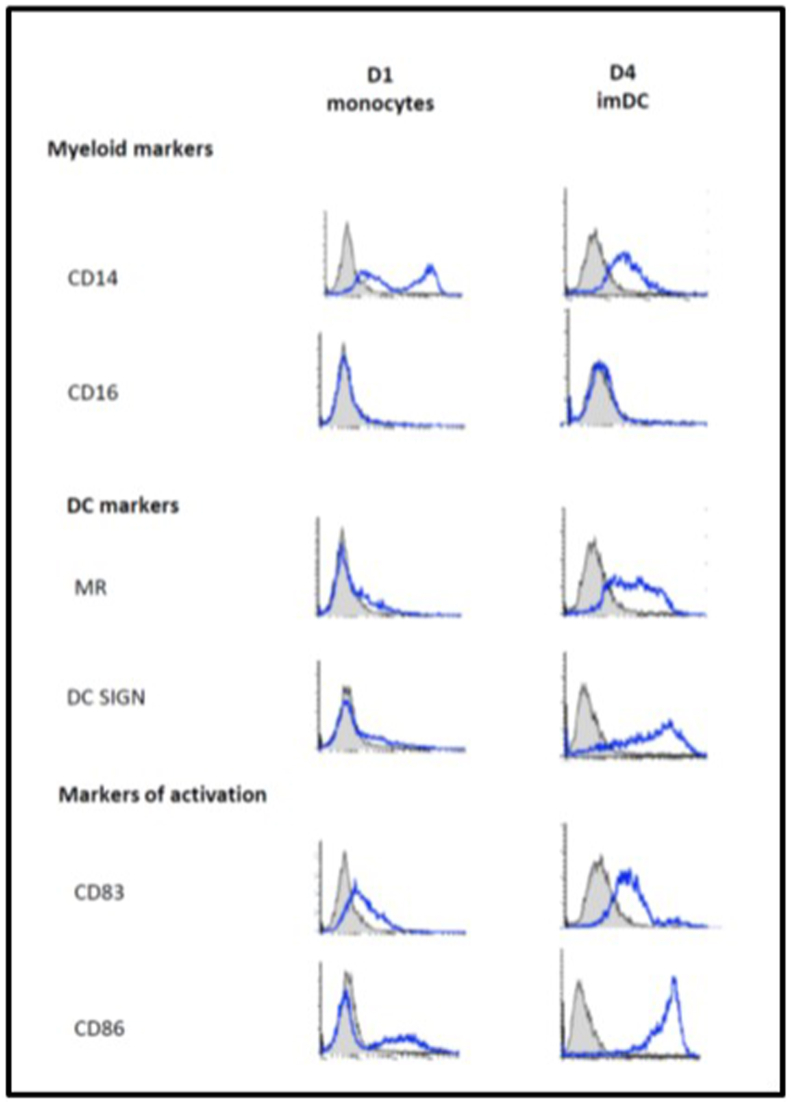
**Phenotyping data for monocyte-derived dendritic cells.** Monocytes were isolated from buffy coats and differentiated into day (D) 4 imDCs. D1 monocytes and D4 imDCs were stained using antibodies to detect 6 different surface markers (CD14, CD16, MR, DC-SIGN, CD83 and CD86). Cells were subjected to flow cytometry and the above histograms demonstrate the expression of these surface markers (in blue) compared to the isotype controls (grey filled). The y-axis represents the number of events and the x-axis the fluorescence intensity. These are representative histograms from two independent experiments. MR; mannose receptor, DC-SIGN; dendritic cell-specific ICAM-3 grabbing non-integrin.

Differentiated imDCs showed increased expression of CD83, CD86, DC-SIGN and mannose receptor (MR) compared to day one monocytes (Figure A1). Previous literature suggests that imDCs do not support antibody dependent enhancement (ADE) in DENV infection (Marovich et al., 2001; Boonnak et al., 2008). Therefore, we confirmed DENV infection of imDCs in a non-ADE setting. The differentiated imDCs were susceptible to DENV infection as suggested by expression of DENV envelope protein (detected with monoclonal antibody 3H5) 48 h following *in vitro* infection with the DENV2 strain 16681 (Fig. 1b). Consistent with productive infection, the DENV envelope protein was expressed in 31 ± 5.5% imDCs; secreted virus was detected in the cell culture supernatant by qRT-PCR at 1.4 × 10^8^ ± 1.7 × 10^7^ genome copies/ml and secreted infectious virus by plaque assay at a titre of 2.4 × 10^5^ ± 3.3 × 10^4^ pfu/ml.

### 3.2 Iminosugars have minimal cytotoxicity in imDCs

All DNJ-derived iminosugars had low cytotoxicity (Table 1), comparable to that seen in primary macrophages after 48 h of treatment (Fischer et al., 1995). E*O*O-DNJ treatment did not show significant cytotoxicity even at a concentration of 1 mM. All iminosugars were used within the non-toxic range (at a concentration below the CC_10_) for further experiments. *N*N-DGJ was used at concentrations not exceeding 31.6 μM due to variable cytotoxicity at higher concentrations; for example, 100 μM resulted in 82.8 ± 31.67% imDC cell viability after 48 h. All other iminosugars were used at concentrations up to 100 μM.

**Table 1:** Antiviral activity and cytotoxicity of iminosugars in imDCs at 48 h post treatment.

Drug	imDC			
	IC_50_, μM (n)	IC_90_, μM (n)	CC_50_, μM (n)	SI
2THO-DNJ	1.6 ± 0.8 (3)	4.7 ± 1.1 (3)	443 (1)	276.87
E*O*O-DNJ	3.1 ± 1.3 (3)	15.4 ± 10 (3)	>1000 (2)	>322.58
*N*N-DNJ	3.3 ± 1.5 (3)	10.1 ± 2.2 (3)	479 ± 211 (2)	145.15
*N*N-DGJ	ND	ND	351 ± 385 (2)	ND

Data shown are mean ± standard deviation. n indicates the number of donors tested.

CC_50_, concentration of iminosugar at which cell viability is 50%; IC_50,_ concentration of iminosugar which inhibits infectious virus secretion by 50%; IC_90_, concentration of iminosugar which inhibits infectious virus secretion by 90%; imDC, immature dendritic cell; ND, not detected (due to the absence of antiviral effects up to 31.6 μM tested); SI, selectivity index.

### 3.3 Iminosugar treatment reduces secretion and specific infectivity of DENV produced in imDCs

To investigate whether iminosugars had an antiviral effect against DENV in imDCs, cells were infected with DENV for 90 min and then treated for 48 h with iminosugars at a serial dose titration from 0.3 to 100 μM. The percentage of infected imDCs reduced with DNJ-based iminosugar treatment in a dose-dependent manner (data not shown). All DNJ-derived iminosugars tested were antiviral, reducing the amount of infectious virus (Fig. 1c) and total number of DENV virions (Fig. 1d) released. In addition, specific infectivity was significantly reduced at iminosugar concentrations ≥31.6 μM (Fig. 1e). In contrast, treatment with <31.6 μM NN-DGJ did not significantly affect the secretion of DENV from imDCs, nor the specific infectivity of secreted virus.

### 3.4 DNJ-derived iminosugar treatment reduces secretion of functional TNFα levels by DENV-infected imDC

**Fig. 2:**
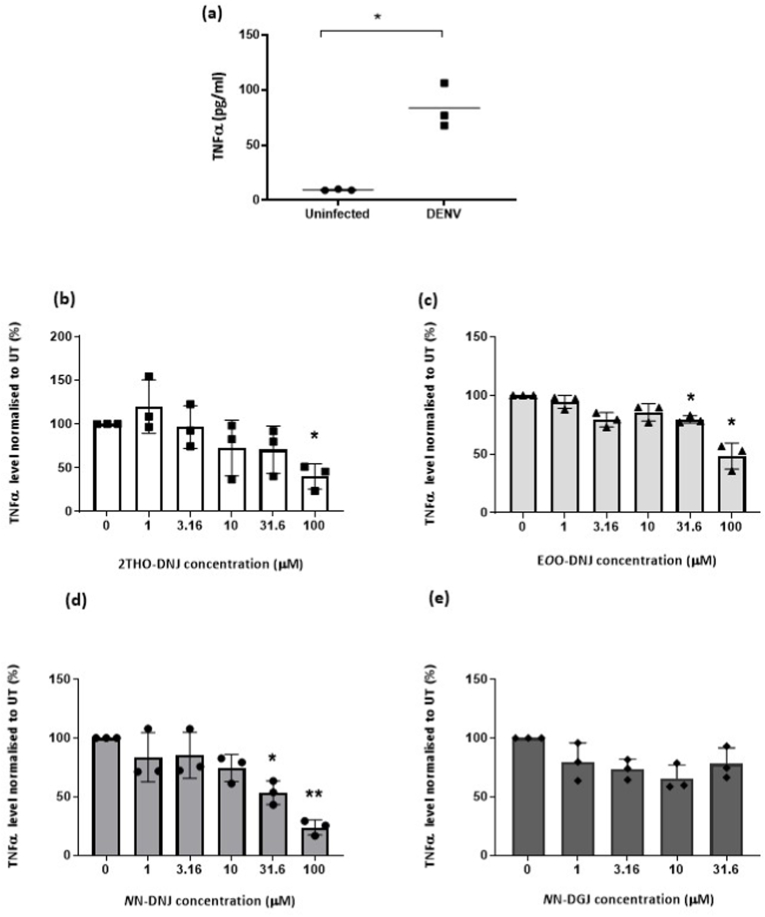
**Functional TNFα levels in DENV-infected imDCs treated with iminosugars.** Primary human imDCs matured from blood-derived monocytes were infected with DENV at a MOI of 1. DENV-infected imDCs were treated with serial titrations of iminosugars for 48 h before the cell supernatant was evaluated for functional TNFα levels by a reporter assay. (a) TNFα secreted by uninfected and DENV-infected imDCs. Secreted TNFα levels in DENV-infected imDCs treated with (b) 2THO-DNJ, (c) E*O*O-DNJ, (d) *N*N-DNJ and (e) *N*N-DGJ. All data are normalized to the untreated (UT) DENV-infected control. Data are derived from three independent experiments. Values represent mean ± SEM; *p < 0.05; **p < 0.01.

Given the important role of TNFα in DENV pathogenesis, we evaluated the effect of iminosugar treatment on TNFα secretion from DENV-infected imDCs. A functional assay was used to ensure that any TNFα detected was biologically functional, as iminosugar treatment could potentially disrupt the folding of the cytokine, a glycoprotein. DENV-infected imDCs secreted functional TNFα (Fig. 2a) and DNJ-derived iminosugars reduced functional TNFα levels in a dose-dependent manner (Fig. 2b–d). Depending on the iminosugar, TNFα levels were significantly reduced at concentrations of 31.6 μM and/or 100 μM. *N*N-DGJ treatment did not reduce functional TNFα levels significantly in DENV-infected imDCs (Fig. 2e).

### 3.5 ER αGluI inhibition by DNJ-derived iminosugars increases the antiviral effect and reduces DENV-induced TNFα levels

**Fig. 3:**
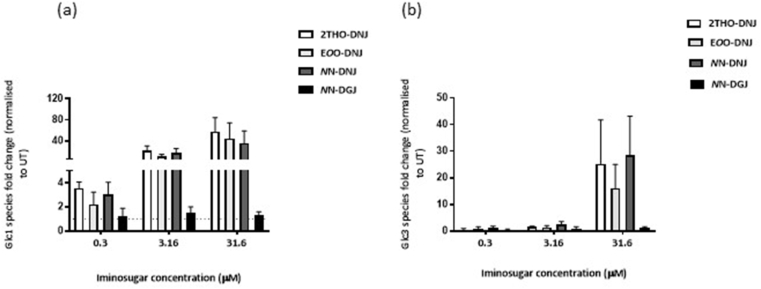
**FOS levels in DENV-infected and iminosugar-treated imDCs.** Primary human imDCs matured from blood-derived monocytes were infected with DENV at a MOI of 1 and treated with 0.3 μM, 3.16 μM and 31.6 μM of the iminosugars 2THO-DNJ, E*O*O-DNJ, *N*N-DNJ and *N*N-DGJ. Cells were harvested at 48 h and FOS levels analysed by NP-HPLC. (a) Glc1 species (resulting from αGluII inhibition) and (b) Glc3 species (resulting from αGluI inhibition) detected in the infected cells are shown. All data are normalized to the untreated (UT) control. Data are derived from three independent experiments. Values represent mean ± SEM.

To better understand the mechanism resulting in the reduction in specific infectivity of DENV and the inhibitory effect of high concentrations of DNJ-derived iminosugars on secreted TNFα levels, iminosugar concentrations at which α-glucosidase inhibition occurs were determined. Accumulation of Glc_1_Man_4_GlcNAc_1_ FOS species occurs due to αGluII inhibition and Glc_3_Man_5_GlcNAc_1_ species are detected following αGluI inhibition (Alonzi et al., 2008). Measurement of FOS derived from DENV-infected imDCs treated with iminosugars revealed that 2THO-DNJ, E*O*O-DNJ and *N*N-DNJ inhibited αGluII at all tested concentrations (Fig. 3a), including 0.3 μM, the concentration corresponding to the onset of reduction in viral secretion. The inhibition of αGluI occurred at 31.6 μM for DNJ-derived iminosugars (Fig. 3b); the concentration at which viral secretion was reduced to less than 20% of untreated samples. Therefore, reductions in both the specific infectivity of secreted virus and functional TNFα levels became apparent at concentrations where αGluI was inhibited. Thus, although αGluII inhibition is sufficient to reduce viral secretion from imDCs, these data suggest that αGluI inhibition could have an additive effect on the antiviral activity and also affect TNFα levels in DENV infection.

## 4 Discussion

To our knowledge, this study provides the first evidence of antiviral effects of DNJ-derived iminosugars in DENV-infected primary human monocyte-derived imDCs, a clinically relevant cell type for natural DENV infection (Cheong et al., 2011; Nakano et al., 2009; Schmid and Harris, 2014; Marovich et al., 2001; Ho et al., 2001). *In vitro* models for antiviral drug evaluation should mimic human infection as closely as possible. Despite limitations of cell culture based experiments, primary cells provide an attractive option to understand cellular responses to infection. Our experiments performed on primary imDCs provide important insights into antiviral and immunomodulatory properties of the host-directed DNJ compounds in DENV infection.

Comparison of the antiviral efficacy of the iminosugars evaluated showed that 2THO-DNJ (IC_50_ 1.6 μM) was more potent than *N*N-DNJ (IC_50_ 3.3 μM) and E*O*O-DNJ (IC_50_ 3.1 μM). *N*N-DGJ did not elicit antiviral effects, consistent with previous literature (Sayce et al., 2016). The absence of α-glucosidase inhibition in imDCs by *N*N-DGJ further supports the conclusion that DNJ-derived iminosugars achieve antiviral effects via ER α-glucosidase inhibition, rather than by inhibition of glycolipid processing (Sayce et al., 2016).

In this study, inhibition of αGluII by DNJ-derived iminosugars was sufficient to induce antiviral effects in imDCs, in keeping with findings for the selective αGluII inhibitor T*O*P-DNJ in MDMΦs (Kiappes et al., 2018). However, key differences were observed in the antiviral effects elicited by iminosugar concentrations inhibiting only αGluII, compared to concentrations at which both α-glucosidases were inhibited. The reduced specific infectivity demonstrates that virions secreted by imDCs treated with iminosugar concentrations inhibiting αGluI have significantly lower infective potential than those produced in imDCs under αGluII inhibition. Iminosugars were previously thought to have insignificant effects on specific infectivity of secreted DENV (Sayce et al., 2016), contrary to the reduced specific infectivity of human immunodeficiency virus (HIV) produced under *N*B-DNJ treatment in the absence of a reduced viral output (Warfield et al., 2020). However, our findings in imDCs suggest that DNJ-derived iminosugars have a dual effect, significantly reducing DENV secretion at all antiviral concentrations, and reducing specific infectivity at concentrations higher than 10 μM which inhibit αGluI. Exploitation of αGluI inhibition provides an attractive option for an enhanced antiviral effect and this concept is further supported by the *in vivo* data available in mice (Miller et al., 2012). Inhibition of αGluI by a single high dose of the iminosugar derivative M*O*N-DNJ (*N*‐(9‐methoxynonyl)‐1‐deoxynojirimycin) prevented death of DENV-infected mice in a lethal antibody-dependent enhanced mouse model even when animals were treated after 48 h post-infection (Miller et al., 2012). These data have important therapeutic implications during dose selection for clinical trials.

In addition to the antiviral effects, iminosugar concentrations inhibiting αGluI were associated with reduced TNFα secretion. The TNFα inhibitory effect seen with iminosugar treatment could be due to the reduction of DENV production by imDCs or due to an independent effect on TNFα secretion. A previous study of M*O*N-DNJ revealed that this DNJ-derivative inhibited the inflammatory response elicited by multiple pathogens and suggested that the effect was modulated via the unfolded protein response (Sayce et al., 2021). The mild reduction of TNFα observed with *N*N-DGJ treatment was not statistically significant or dose-dependent as expected with this compound. The current paper does not attempt to understand the exact mechanism of the reduction in TNFα production observed with iminosugar treatment in imDCs, but experiments designed to answer this question are of interest for the future.

## 5 Conclusions

The DNJ-derived iminosugars *N*N-DNJ, 2THO-DNJ and E*O*O-DNJ reduce secretion of virus from DENV-infected imDCs in a dose-dependent manner. In addition, at concentrations inhibiting αGluI, these compounds reduce the infectivity of the virus that is released, and also reduce TNFα secretion. Taken together, these data suggest that αGluI inhibition by DNJ-derived iminosugars mediates important antiviral effects in DENV-infected imDCs, beyond the reduction in viral secretion achieved with αGluII inhibition alone.

## Funding

This work and author JLM was funded by the Oxford Glycobiology endowment. NP was funded by the Commonwealth Scholarship Commission. JB and BET were supported by the 10.13039/100010269Wellcome Trust [grant numbers 203853/Z/16/Z and 105402/Z/14/Z, respectively].

## Declaration of competing interest

Authors NP, JB, DA, BET, JLM and NZ declare that they have no known competing financial interests or personal relationships that could have appeared to influence the work reported in this paper.
